# A Multimodal Curriculum With Patient Feedback to Improve Medical Student Communication: Pilot Study

**DOI:** 10.5811/westjem.2018.11.44318

**Published:** 2019-12-09

**Authors:** Nicole M. Dubosh, Matthew M. Hall, Victor Novack, Tali Shafat, Nathan I. Shapiro, Edward A. Ullman

**Affiliations:** * Beth Israel Deaconess Medical Center/Harvard Medical School, Department of Emergency Medicine, Boston, Massachusetts; † Soroka University Medical Center and Faculty of Health Sciences, Ben-Gurion University of the Negev, Beer-Sheva, Israel

## Abstract

**Introduction:**

Despite the extraordinary amount of time physicians spend communicating with patients, dedicated education strategies on this topic are lacking. The objective of this study was to develop a multimodal curriculum including direct patient feedback and assess whether it improves communication skills as measured by the Communication Assessment Tool (CAT) in fourth-year medical students during an emergency medicine (EM) clerkship.

**Methods:**

This was a prospective, randomized trial of fourth-year students in an EM clerkship at an academic medical center from 2016–2017. We developed a multimodal curriculum to teach communication skills consisting of 1) an asynchronous video on communication skills, and 2) direct patient feedback from the CAT, a 15-question tool with validity evidence in the emergency department setting. The intervention group received the curriculum at the clerkship midpoint. The control group received the curriculum at the clerkship’s end. We calculated proportions and odds ratios (OR) of students achieving maximum CAT score in the first and second half of the clerkship.

**Results:**

A total of 64 students were enrolled: 37 in the control group and 27 in the intervention group. The percentage of students achieving the maximum CAT score was similar between groups during the first half (OR 0.70, p = 0.15). Following the intervention, students in the intervention group achieved a maximum score more often than the control group (OR 1.65, p = 0.008).

**Conclusion:**

Students exposed to the curriculum early had higher patient ratings on communication compared to the control group. A multimodal curriculum involving direct patient feedback may be an effective means of teaching communication skills.

## INTRODUCTION

The doctor-patient relationship is deemed one of the most important aspects of a medical encounter. Effective communication has clear benefits to both the patient and the provider. Patients who perceive their healthcare providers as strong communicators tend to have better expectations of their healthcare course, adhere to positive health behaviors, and report higher satisfaction.^1^–^4^ For physicians, effective communication correlates with more positive patient interactions, decreased risk of litigation, and decreased burnout.^5^,^6^ Effective communication can be particularly challenging in the emergency department (ED) given the chaotic environment, time and resource constraints, and lack of continuity of care. In a prospective observational study, only two-thirds of emergency physicians discussed ED course and necessary follow-up with their patients and patients frequently misunderstood information conveyed by their provider.^7^ An emphasis on fostering communication skills in the emergency medicine (EM) clerkship may improve this competency.

There is an increased focus on interpersonal skills and communication in medical education.^8^ The Association of American Medical College has revised core competencies, and the Accreditation Council for Graduate Medical Education (ACGME) Entrustable Professional Activities for entering residency include interpersonal and communication skills.^9^,^10^,^11^ While many medical schools include specific courses on patient-centered communication during the preclinical years, there is often a lack of dedicated teaching on this topic during the clinical clerkships. Two studies demonstrated a decline in medical students’ interpersonal skills and patient-centered attitudes from the first through fourth year.^12^,^13^ A dedicated curriculum during the clinical years may help improve students’ communication skills and prevent this decline.

To address the need of improving our educational approach for physicians-in-training on effective communication, we developed and implemented a novel, multimodal curriculum incorporating direct patient feedback to teach and assess this competency in the EM clerkship. The objective of this study was to assess whether a multimodal curriculum including direct patient feedback improves medical student communication skills as measured by the Communication Assessment Tool (CAT).

## METHODS

### Study Design and Setting

This was a prospective, randomized, pilot study. Our study was reviewed by the institutional review board at our institution and was determined to be exempt. We included all students enrolled in the fourth-year EM clerkship from July 2016–October 2017. The study institution is an urban, tertiary care, Level 1 trauma center with an ED census of 55,000 patients annually and home to a three-year EM residency program.

### Study Protocol

We developed a multimodal curriculum to teach communication skills consisting of two parts: 1) an asynchronous video on communication skills; and 2) delivery of direct patient feedback from the CAT questionnaire to the student. We designed this curriculum using principles of curricular development described by Kern.^14^ Through our needs assessment based on faculty evaluations, verbal nursing comments, and observation during simulation, we identified that students’ communication skills are extremely variable. Furthermore, medical students routinely do not receive direct patient feedback. Our goal was to develop a curriculum that would expose our targeted learner group, fourth-year medical students in EM, to this critical aspect of patient care and determine its utility in teaching and assessing communication skills in this population. To add framework to our curriculum, we included a video module based on prior work that has demonstrated efficacy of asynchronous curricula compared to traditional synchronous didactics.^15^,^16^ We then sought to implement and prospectively assess our curriculum by looking at patient ratings of communication skills.

**Educational Research Capsule Summary**

What do we already know about this issue?

*Effective communication is essential for the doctor-patient relationship, yet dedicated education and assessment strategies are lacking*.

What was the research question?

Does a multimodal curriculum including direct patient feedback improve medical student communication in an emergency medicine clerkship?

What was the major finding of the study?

*Students exposed to the curriculum showed improved patient ratings on communication abilities*.

How does this improve population health?

*Medical educators should consider a curriculum involving patient feedback as a means of teaching effective communication skills. This may in turn improve patient care*.

The undergraduate medical education team designed the video that was made available online for student access. It is approximately 13 minutes long and includes evidence-based content on the importance of effective patient-doctor communication, barriers, and techniques for success. The format of the video includes narrated slides and structured interviews from EM academic faculty and the social work team. Faculty invited to participate in the video were those who consistently received the highest teaching scores by medical students and residents.

To assess medical student communication skills, we used the CAT along with free-response comments from patients (see Appendix 1). The CAT is a 15-item questionnaire that assesses communication skills from the patient perspective and has validity evidence to support its use. The questions use a 1–5 rating scale with 1 being “poor” and 5 being “excellent” and cover multiple domains related to communication and interpersonal skills.^17^ It has demonstrated utility in assessing communication skills in surgery and family medicine residents.^18^,^19^ The CAT has also been administered to ED patients and captures the patient’s perspective on the overall team’s communication skills.^20^ Its utility in assessing medical student communication skills has not yet been studied, nor have any other patient communication assessment tools been shown to have validity evidence in the medical student population. Because the last question of the CAT pertains to the communication skills of the entire ED team, we omitted this item and calculated student CAT scores out of 70 points for the remaining 14 questions based on previous approaches.^20^

During the study period, trained research assistants (RA) administered the CAT survey and free-response questions to ED patients cared for primarily by a fourth-year clerkship student. We implemented a system whereby a text page notification was sent to the RA team when a student signed up for a patient on our ED’s electronic tracking board. Pages were sent during the hours of 8 am–11 pm Monday through Friday and every odd weekend day when the RA staff was available. We included patients if they could identify the medical student who cared for them by photo, did not require interpreter services, and were at baseline alert and orientated to person, place and time. Only discharged patients were included in accordance with our institution’s policy regarding patient surveys.

The RA informed the patient that the purpose of the survey was to help the student better his or her communication skills. Written consent was obtained from eligible patients for the use of their de-identified survey data for research purposes. We field-tested the administration of the CAT questionnaire during the month prior to the start of the study as a training period for RAs and to ensure adequate selection of patients. In response to this field testing, we made changes specifically regarding the timing of the pages sent to the RAs in order to maximize the number of patients screened prior to discharge.

To study the effect of our curriculum, we assigned students into an intervention group or control group. Students were randomized based on clerkship month such that all students rotating in the department received the educational experience. Group assignment alternated every other month (ie, all students in July received the curriculum mid-month while all students in August received the curriculum at the end of the clerkship). All students were notified at the beginning of the clerkship that we were instituting a new communication curriculum involving collection of patient feedback. The students in the intervention group were assigned to watch the video at the end of the second week of the clerkship at which time they also were given their CAT scores and free-response patient comments from the first two weeks of the clerkship.

The clerkship directors delivered the patient’s feedback to the medical student in a face-to-face meeting. Additionally, the clerkship directors discussed with them ways to improve these skills. Students in the control group were assigned to watch the video at the end of the fourth week of the clerkship and received feedback from the CAT and patient comments for the entire four-week clerkship at that time (Figure). students in both groups were required to watch the video as part of the required clerkship curriculum. They were asked to verify they had viewed it via an email survey of confirmation.

### Outcome Measures

We compared CAT patient questionnaire ratings for students in the intervention vs control groups during the first and second halves of the clerkship. Free-response comments from patients regarding their medical student’s communication skills were also collected. Additionally, we assessed via our standard end-of-clerkship survey whether or not the students had ever received direct patient feedback previously in their medical school training. Student and patient participation in the study was voluntary. Students provided written consent for the use of their de-identified data for research purposes. By completing the survey, patients gave verbal consent for use of their de-identified data.

### Outcome Measures and Data Analysis

CAT scores and free-response patient comments were de-identified and recorded in a REDCap database^21^ that was stored on a secure server. Prior studies using the CAT demonstrate that a dichotomized scoring system was more useful than mean score given the ceiling effect (ie, there is an inherent skewing of mean scores toward the upper end of the 5-point scale).^17^, ^20^, ^22^ Given this, we dichotomized the total score into maximal score (70 points) and sub-maximal (less than 70) as has been done previously. Categorical data were expressed as absolute numbers and percentages, and parameters with non-parametric distribution as median and interquartile range.

Differences in CAT scores between the intervention and control groups were assessed by Mann Whitney test for variables with non-parametric distribution and chi-square test (x^2^) for categorical variables. We used generalized estimating equation logistic regression model (unstructured matrix) to compare proportions of maximal CAT score (score = 70) between intervention vs control group. This accounts for the clustering of the responses by the same medical student, questionnaires at baseline (weeks 1–2 of the clerkship) and after intervention (weeks 3–4 of the clerkship). This statistical approach allows for adjustment of the results given the variability in number of CAT questionnaires per student and adjusts to the correlation between the different interviews of the same subject. This helps to achieve an unbiased estimate in the following hypothetical situation: one or more students in the intervention group is extremely responsive to the training and also has more questionnaires than others. P-values < 0.05 were considered statistically significant. We also calculated the percentage of students who reported receiving direct patient feedback previously in medical school. All statistical analyses were conducted using SPSS 25.0 (IBM Corp Armonk, NY).

## RESULTS

We enrolled 64 students during the study period: 37 in the control group and 27 in the intervention group. All students confirmed they had watched the video. There were no major differences among gender, home vs visiting students, and percentage of students applying to EM between groups (Table 1). A total of 321 CAT questionnaires were administered. The median number of questionnaires per student was five. In the first half of the clerkship, the percentage of students with the maximum CAT score was similar between the intervention and control groups: 57.5% and 59.7%, respectively. In the second half of the clerkship, students in the intervention group achieved a maximum score more often than the control group: 62.3% and 51.1%, respectively.

In the logistic regression model, prior to the intervention (weeks 1–2), there was no difference between the groups (odds ratio (OR) [0.70], 95% confidence interval (CI), 0.44–1.13, p = 0.148). During the second half of the clerkship (weeks 3–4), the intervention group students achieved a maximum score more often than the control group (OR [1.65], 95% CI, 1.14–2.41, p = 0.008, Table 3). Representative patient feedback comments are displayed in Table 2. On the post-clerkship survey, 27% of students in our study reported receiving patient feedback previously in medical school.

## DISCUSSION

We demonstrated successful deployment of a multimodal curriculum consisting of an asynchronous online video coupled with direct patient feedback to teach and assess student communication skills in an EM clerkship. To the best of our knowledge, this is the first dedicated curriculum that incorporates direct patient feedback in the clinical clerkship years.

It is interesting to note that while there was an increase in CAT scores in the intervention group during the study period, there was an overall decrease in the control group. It is difficult to discern the reason for the drop in CAT scores in the control group during the study period. One possibility is the decline parallels the trend that has been previously demonstrated in interpersonal skills across the duration of medical school.^12^,^13^ It is possible that at baseline all students do indeed have a decrease in communication skills over the month of a clerkship and that our curriculum mitigated this decline in the intervention group. Alternatively, this decline may have been due to a sampling error given the relatively small study population.

Undergraduate medical education curricula for teaching communication skills typically use traditional teaching modalities. Systematic, standardized techniques such as the Calgary-Cambridge Observation Guide and CLASS protocol have been previously used for framing the patient interview with a focus on optimizing communication.^23^–^25^ Simulation is widely employed as an educational modality to improve learners’ communication skills and has demonstrated feasibility through learner self-assessment surveys.^26^,^27^ Rucker et al. developed a longitudinal communication curriculum for medical students consisting of seminars and videotaped interactions. After initiation of this curriculum, students’ communication scores improved significantly on an objective structured clinical examination (OSCE).^28^ The findings of our study add to the existing literature by offering another potential educational modality for teaching communication skills in the clerkship years.

In terms of assessment of communication skills, standardized patients and direct observation are commonly used modalities in EM students,^23^ and there is substantial evidence demonstrating their feasibility^29^,^30^ There are some limitations, however, with their day-to-day use. Standardized patients often require substantial scheduling efforts, nonclinical workspace, and monetary cost. These modalities may also introduce observer bias as the perception of the interaction is not made by the primary participants of the doctor–patient relationship. While the OSCE is an important means of evaluation, it still suffers from variability of rater scales.^31^ Using the patient as the assessor may lessen the resource utilization and funding needs often required of these more traditional modalities. It also allows for more distinct evaluative encounters, which thereby may increase feedback. While our study used RAs, an ED attending, nurse, or tech could easily administer the CAT, as the approximate amount of time spent to administer the survey was five minutes. This strategy could avoid the extra cost of RAs and therefore allow this program to be more feasibly implemented. The breakdown of time and monetary costs can be found in Appendix 2.

On a broader scale, there is limited data regarding the use of direct patient feedback in improving communication skills in EM. In a recent prospective, randomized, pilot study of EM attending physicians, an intervention using monthly email feedback and face-to-face meetings on Press-Ganey scores did not improve provider patient-satisfaction scores compared to the control group.^32^ These findings are in direct contrast with our results. Reasons for this are unclear, but there are inherent differences in the content assessed by Press-Ganey and the CAT as well as differences in motives for using these tools that may contribute. Further studies are needed to assess the effect of patient feedback on clinicians across all levels of training and practice. What is surprising is that in our post-clerkship survey, the overwhelming majority of students in our study (73%) had never received direct patient feedback in their medical school training up to this point, making our approach novel. This further highlights the potential role for this type of curriculum in undergraduate medical education.

Perhaps one of the more interesting aspects of our curriculum is the ability for incorporation into a 360-degree student evaluation. Prior studies have demonstrated successful implementation of multi-source, workplace-based assessment programs including patient feedback in various clinical settings.^33^,^34^ The data on whether or not these lead to improved performance is mixed, although such programs generally receive positive ratings by physicians.^35^ The ACGME has suggested the use of multi-source feedback and multiple evaluators for assessing trainees’ competencies across multiple domains.^36^ As healthcare continues to move toward a patient-centered view, this is critical to the development of future physicians. In a prospective study of pediatric residents, faculty and nurses rated the trainees higher on professionalism and interpersonal skills than did patients and families.^37^ Further investigations are needed to determine how patient ratings compare to those of faculty and other healthcare providers. Including the patients’ view in student evaluations may add depth to the feedback and specific focus for improvement.

## LIMITATIONS

First, this was a proof-of-concept, single-center study with a relatively small sample size that may limit extrapolation to other institutions. We believe, however, that the fact that medical students in our study come from 31 different medical schools adds heterogeneity to our population and may enhance generalizability. Second, only patients who were discharged from the ED were included in our study, as we did not want to affect the Health Care Consumer Assessment of Healthcare Providers and Systems survey administration to admitted patients.^38^ This skews our patient population to those who are lower acuity, and therefore we cannot draw conclusions about medical student communication in the higher-acuity patient population. Third, the inherent ceiling effect (the nature of patients being surveyed to give high scores) we see with the CAT scores may further minimize differences between groups.

Fourth, due to the one-month nature of the clerkship, the post-intervention measures were collected immediately after the curriculum was delivered to the intervention group. A future study in which post-intervention CAT scores are collected at a later time is needed to assess for a washout effect. Fifth, it is possible that the Hawthorne effect may have contributed both in terms of student performance and patient responses. We attempted to minimize such effect in terms of student performance by notifying all students at the beginning of the clerkship that we would be gathering patient feedback. Finally, because our curriculum is multimodal, we could not discern the extent to which the patient feedback, the video module, or the feedback discussion session with the clerkship directors had effect on the observed outcome.[Table t4-wjem-21-115]

## CONCLUSION

A multimodal curriculum incorporating asynchronous learning and direct patient feedback is a feasible modality for teaching and assessing medical student communication skills in a fourth-year EM clerkship. Students in the intervention group attained higher patient ratings on communication skills compared to the control group. Undergraduate medical educators should consider using this novel approach in teaching and assessing communication and interpersonal skills.

## Supplementary Information





## Figures and Tables

**Figure:**
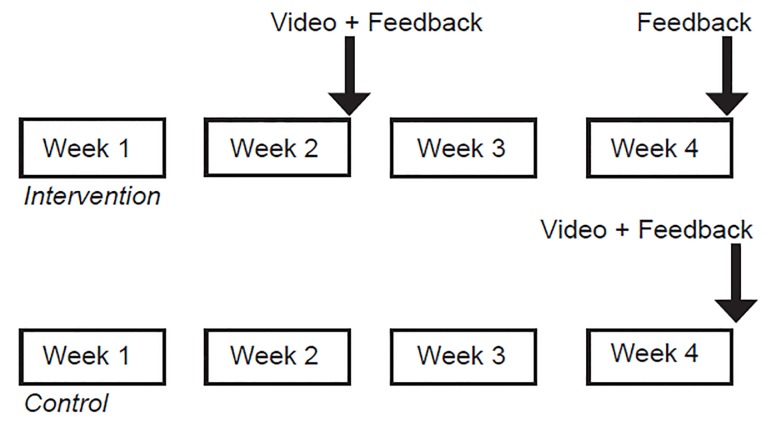
Multimodal communication curriculum for emergency medicine clerkship students for intervention versus control groups.

**Table 1:** Demographic characteristics of medical students in the emergency medicine clerkship multimodal communication curriculum.

	Intervention (n=27)	Control (n=37)
Male, n (%)	16 (59)	22 (59)
Home institution medical students, n (%)	8 (30)	12 (32)
Visiting medical students, n (%)	19 (70)	25 (68)
Number of medical schools represented	16	22
Students applying to EM, n (%)	19 (70)	26 (70)

*EM*, emergency medicine.

**Table 2:** Univariate analysis of intervention group vs control group.

	Total (n students =64, n questionnaires=321)	Intervention (n students =27, n questionnaires=150)	Control (n students=37, n questionnaires=171)	P value
Questionnaires per student (Median, IQR)	5 (3–7)	5 (3–7)	4 (3–6)	0.202
Questionnaires with maximal score at baseline (weeks 1–2) (n, %) among total questionnaires (n)	88 (58.7) (n=150)	42 (57.5) (n=73)	46 (59.7) (n=77)	0.784
Questionnaires with maximal score after intervention (weeks 3–4) (n, %) among total questionnaires (n)	96 (56.1) (n=171)	48 (62.3) (n=77)	48 (51.1) (n=94)	0.139

*n*, number; *IQR*, interquartile range.

**Table 3:** Odds ratio for maximal Communication Assessment Tool score (score = 70) for intervention versus control group questionnaires, at baseline (weeks 1–2) and after intervention (weeks 3–4).

	OR	95% CI	P value
Weeks 1–2	0.70	0.44–1.13	0.148
Weeks 3–4	1.65	1.14–2.41	0.008

*CI*, confidence interval; *OR*, odds ratio.

**Table 4 t4-wjem-21-115:** Representative patient free-response comments on emergency medicine clerkship students’ communication skills.

“Appreciate how personable he was. He could elaborate more when he comes to the follow-up information.”
“He was very attentive and took time to explain things clearly.”
“He was good. But really the attending doctor gave me much more detailed information.”
“She has good communication skills, she is very friendly, and she has a general concern for helping patients.”
“She didn’t give me all of the information I wanted to know. She seemed very nervous and a bit uncomfortable.”
“She was excellent. At first I was unsure about a med student, but she actually spent a lot of time with me. She was very thorough and is an excellent physician.”
“I had felt very upset about my accident, and she made me feel much better. She legitimized my concerns and feelings 100%.”
“She listened attentively.”
“My suggestion would be to make sure that any information he has or knows is explained to me, the patient.”
