# Supplementary material for: A Multimodal Curriculum With Patient Feedback to Improve Medical Student Communication: Pilot Study
Source: West J Emerg Med. 2019 Dec 9;21(1):115–21. doi: 10.5811/westjem.2018.11.44318 (PMC6948689; doi:10.5811/westjem.2018.11.44318)
Supplement: Supplementary file 1 [file wjem-21-115-s001.docx]

**Appendix 1:** Communication Assessment Tool


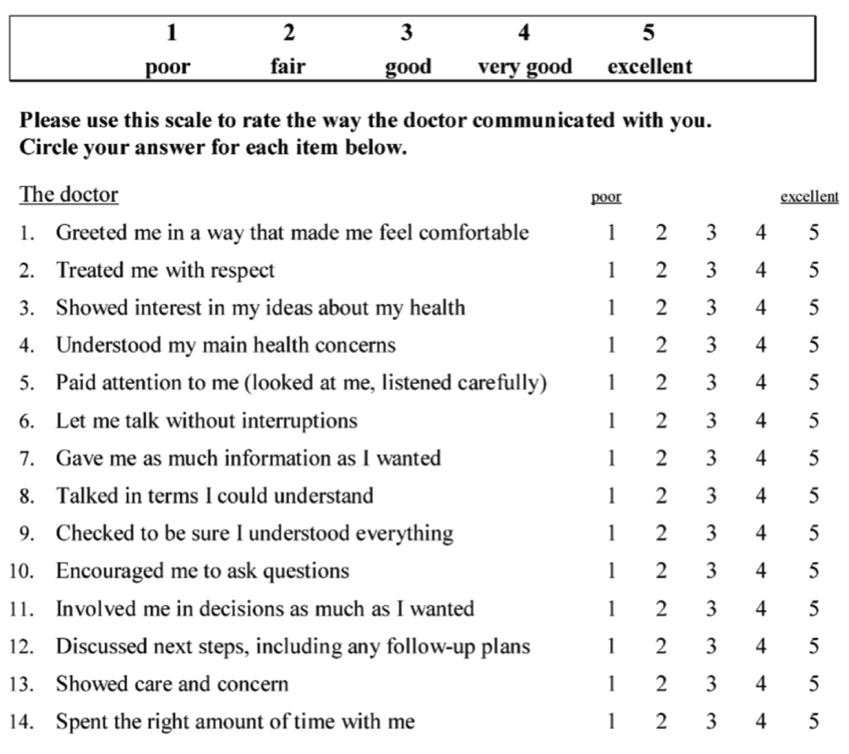


Adapted from Makoul G, Krupat E, Chih-Hung C. Measuring patient views of physician communication skills: development and testing of the communication assessment tool. *Patient Educ Couns.* 2007;67:333-342.
