# Supplementary material for: A Multimodal Curriculum With Patient Feedback to Improve Medical Student Communication: Pilot Study
Source: West J Emerg Med. 2019 Dec 9;21(1):115–21. doi: 10.5811/westjem.2018.11.44318 (PMC6948689; doi:10.5811/westjem.2018.11.44318)
Supplement: Supplementary file 2 [file wjem-21-115-s002.docx]

**Appendix 2: Time and Monetary Cost Breakdown**

| Clerkship director approximate time to deliver feedback, per student | 5 mins |
| --- | --- |
| RA approximate time to administer CAT questionnaire, per patient | 5 minutes |
| Total RA time spent for 321 CAT questionnaires | 1605 minutes (26.75 hours) |
| Total RA cost for CAT questionnaire administration | $802.50 (RA hourly cost, $30.00) |

Abbreviations: RA indicates research assistant, CAT indicates Communication Assessment Tool
